# Long-term impact of the COVID-19 pandemic on the quality of life of people with dementia and their family carers

**DOI:** 10.1093/ageing/afad233

**Published:** 2024-01-25

**Authors:** Sanna Read, Ben Hicks, Emily Budden, Jacob Douglass, Amanda Grahamslaw, Elena Herrero, Gregory Joseph, Christine Kirkup, Martha Pusey, Alice Russell, Harsharon Sondh, Sharon Sondh, Bryony Storey, Georgia Towson, Kate Baxter, Yvonne Birks, Carol Brayne, Carmen Colclough, Margaret Dangoor, Josie Dixon, Paul Donaghy, Kate Gridley, Peter R Harris, Bo Hu, Derek King, Martin Knapp, Eleanor Miles, Christoph Mueller, Rotem Perach, Louise Robinson, Jennifer Rusted, Alan J Thomas, Raphael Wittenberg, Sube Banerjee

**Affiliations:** Care Policy and Evaluation Centre, London School of Economics and Political Science, London, UK; Brighton and Sussex Medical School, Centre for Dementia Studies, University of Sussex, Brighton, UK; Sussex Partnership NHS Foundation Trust, Worthing, UK; Gateshead Health NHS Foundation Trust, Gateshead, UK; Gateshead Health NHS Foundation Trust, Gateshead, UK; South London and Maudsley NHS Foundation Trust, London, UK; South London and Maudsley NHS Foundation Trust, London, UK; Gateshead Health NHS Foundation Trust, Gateshead, UK; Sussex Partnership NHS Foundation Trust, Worthing, UK; Sussex Partnership NHS Foundation Trust, Worthing, UK; South London and Maudsley NHS Foundation Trust, London, UK; South London and Maudsley NHS Foundation Trust, London, UK; Gateshead Health NHS Foundation Trust, Gateshead, UK; Sussex Partnership NHS Foundation Trust, Worthing, UK; Social Policy Research Unit, Faculty of Social Sciences, University of York, York, UK; Social Policy Research Unit, Faculty of Social Sciences, University of York, York, UK; Cambridge Public Health, University of Cambridge, Cambridge, UK; School of Psychology, University of Sussex, Brighton, UK; Care Policy and Evaluation Centre, London School of Economics and Political Science, London, UK; Care Policy and Evaluation Centre, London School of Economics and Political Science, London, UK; Kings College London, Institute of Psychiatry, London, UK; Social Policy Research Unit, Faculty of Social Sciences, University of York, York, UK; School of Psychology, University of Sussex, Brighton, UK; Care Policy and Evaluation Centre, London School of Economics and Political Science, London, UK; Care Policy and Evaluation Centre, London School of Economics and Political Science, London, UK; Care Policy and Evaluation Centre, London School of Economics and Political Science, London, UK; School of Psychology, University of Sussex, Brighton, UK; Kings College London, Institute of Psychiatry, London, UK; School of Psychology, University of Sussex, Brighton, UK; Population Health Sciences Institute, Newcastle University, Newcastle, UK; School of Psychology, University of Sussex, Brighton, UK; Institute for Ageing, Newcastle University, Newcastle, UK; Care Policy and Evaluation Centre, London School of Economics and Political Science, London, UK; Faculty of Health, University of Plymouth, Plymouth, UK

**Keywords:** quality of life, dementia, carers, COVID-19, cohort, older people

## Abstract

**Introduction:**

Few studies have longitudinally mapped quality of life (QoL) trajectories of newly diagnosed people with dementia and their carers, particularly during coronavirus disease-2019 (COVID-19).

**Methods:**

In a UK cohort study, 261 newly diagnosed people with dementia and 206 family carers were assessed prior to the pandemic (July 2019–March 2020), followed up after the first lockdown (July–October 2020) and then again a year and 2 years later. Latent growth curve modelling examined the level and change of QoL over the four time-points using dementia-specific QoL measures (DEMQOL and C-DEMQOL).

**Results:**

Despite variations in individual change scores, our results suggest that generally people with dementia maintained their QoL during the pandemic and experienced some increase towards the end of the period. This contrasted with carers who reported a general deterioration in their QoL over the same period. ‘Confidence in future’ and ‘Feeling supported’ were the only carer QoL subscales to show some recovery post-pandemic.

**Discussion:**

It is positive that even during a period of global disruption, decline in QoL is not inevitable following the onset of dementia. However, it is of concern that carer QoL declined during this same period even after COVID-19 restrictions had been lifted. Carers play an invaluable role in the lives of people with dementia and wider society, and our findings suggest that, post-pandemic, they may require greater support to maintain their QoL.

## Key Points

- People with dementia maintained their life quality despite a global pandemic.
- Carers quality of life declined during this same period, even after pandemic restrictions were lifted.
- Family carers may require greater formal support to sustain their life quality after the pandemic.

## Introduction

Dementia is characterised by progressive decline in cognition that interferes with social and/or occupational functioning [1, 2]. One of the great healthcare challenges of the 21st century, supporting the estimated 55 million people worldwide with the condition, is an international health policy priority [3–5].

Quality of life (QoL), widely used in research examining experiences of the general population as well as in dementia [6, 7], is a multidimensional construct including physical health, psychological state, social relationships, personal beliefs, level of independence and environmental support [7]. Modifiable elements may differ for and between people with dementia [6, 8, 9] and family carers [7, 10] that can positively or adversely impact QoL. These factors include demographic (e.g. spouse/non-spouse carer, living situation), personal (e.g. coping strategies), social (e.g. familial networks) and contextual (e.g. ability to contribute to their community). Longitudinal studies examining changes in QoL for people with dementia typically report that QoL, rated by the person with dementia, remains stable over time and over the course of the condition, suggesting deterioration of QoL may not be an inevitable part of dementia progression. This contrasts with carer/proxy ratings of people with dementia’s QoL that tend to decrease over time and across the stages of the condition [11–14]. Longitudinal studies of carers of people with dementia suggest that their own QoL also remains stable over periods of up to 3 years [15–18]; although Valimaki et al. [18] showed carers of people with dementia start with lower QoL (compared to an age- and gender-standardised general population) and dementia severity may have negative impact on mood and burden-related dimensions of carer QoL, if not overall scores. There are issues concerning participation in research, the nature of the recruitment source as well as methodological issues regarding measurement of QoL in these studies, with none to date using tools specifically designed to assess QoL in dementia populations. We aimed to add to this limited evidence base by longitudinally examining, across multiple time-points, the QoL of people recently diagnosed with dementia and their carers using dementia-specific QoL measures.

During the pandemic within which this study was completed, it was reported that the QoL of people with dementia and/or their carers decreased in the short to mid-term [19–25]. No study has examined the on-going QoL of people with dementia and their carers post-pandemic to examine changes following restoration of services and informal and formal social support. We aimed to address this by examining QoL trajectories of a cohort of people newly diagnosed with dementia in the months before the first coronavirus disease-2019 (COVID-19) outbreak and investigating how these trajectories varied using multiple follow-ups and examining the different dimensions of QoL. We hypothesised that QoL for people with dementia would be maintained or improved following the removal of COVID-19 restrictions and declines in carer QoL, previously noted [25], would be reversed.

## Methods

### Sample

The DETERMIND cohort [26] includes people with dementia clinically diagnosed within 6 months of baseline assessment, and their carers, in three areas of England (North-East, South London and Sussex). Recruited via multiple routes, 261 people with dementia and 206 carers were comprehensively assessed prior to the pandemic at baseline (T1 July 2019 to March 2020) and followed up in July–October 2020 (DETERMIND-C19 additional study) after the first COVID-19 lockdown in England. DETERMIND-C19 interviews were conducted by telephone; all carers were eligible to participate but only people with dementia with capacity to provide informed consent at T1 were approached. Since then, participants were contacted for re-interview a year (T2) and 2 years (T3) later. Most T2 and T3 interviews were conducted face-to-face once restrictions were lifted. Attrition rates for each wave of data collection are reported in the results. Ethics approvals for DETERMIND and DETERMIND-C19 were granted by the Health Research Authority Brighton and Sussex Research Ethics Committee (REC 19/LO/0528. IRAS 261263).

### Measures

We measured self-rated and carer-rated QoL of people with dementia using the 28-item DEMQOL (range 28–112) and 31-item DEMQOL-Proxy (range 31–124) [27]. These interviewer-administered, dementia-specific questionnaires assess health-related QoL over the previous week. We calculated total and subscale (Feelings, Memory and Everyday life) scores. To assess carer QoL, we used the 30-item C-DEMQOL (range 30–150) [28], an interviewer-administered, dementia-carer-specific questionnaire assessing QoL over the past 4 weeks. We calculated total scores and scores on six subscales (Meeting personal needs, Carer wellbeing, Carer-patient relationship, Confidence in future, Feeling supported).

Variables measured included demographic and other characteristics associated with QoL for people with dementia and/or carers [6, 7, 29, 30], including study site, co-residence of person with dementia/carer, age, gender, marital status, education (level of qualifications attained), occupational class based on the National Statistics Socio-economic Classification (NS-SEC), work status, home ownership, Office of National Statistics (ONS) rural–urban classification of postcode area [31], deciles of Index of Multiple Deprivation (IMD) based on postcodes [32] and number of months since diagnosis of dementia and between baseline and C19 interview. Cognitive impairment was measured using the Mini-Mental State Examination (MMSE) [33]; Clinical Dementia Rating (CDR) [34] scores were available for people with dementia at all time-points who had a carer consented into DETERMIND with an index score computed using the National Alzheimer’s Coordinating Centre online calculator.

### Analysis

Latent growth curve (LGC) modelling examined the level and change over four time-points in QoL and associations with characteristics of the carer and person with dementia using Mplus8 [35]. In a LGC model [36], random effects measure individual differences and fixed effects estimate the average growth of the entire sample. Piecewise LGCs were fitted to investigate non-linear patterns and specific time periods: before and after the first lockdown (T1–C19), during the pandemic (C19–T3) and over the last lockdown and vaccine campaign (T2–T3). Participant characteristics collected at baseline were included as potential predictors of level and change. For DEMQOL-Proxy models (carer rated), carer education and occupational status were included because they were associated with DEMQOL-Proxy scores. CDR score was used as a time-varying covariate. The uneven time between the first two measurement occasions (T1–C19) was adjusted for the number of months between the baseline and C19.

Model fit was assessed by Chi-square analysis (*P* > 0.05 interpreted as good fit), but as this is sensitive to sample size [37], we used three other recommended fit indices [38]: comparative fit index (CFI), root mean square error of approximation (RMSEA) and standardised root mean square residual (SRMR). Values at or below 0.080 for RMSEA and SRMR and at or above 0.90 for CFI indicate adequate model fit. Maximum likelihood estimation with robust standard errors was used to handle sample non-normality. Full information maximum likelihood method [39] was used to include cases with partially missing values in path models so that information on means and variances of all data are used (see Supplementary text 1 for attrition).

## Results

### Descriptive

Table 1 presents characteristics of carers and people with dementia; average age at baseline was 66 for carers and 80 for people with dementia. Most carers were female (69%), married (82%) and co-resident (67%) with the person with dementia. At baseline, 72% of people with dementia scored >19 on the MMSE (mild impairment). Mean time from diagnosis of dementia (62% Alzheimer’s disease, 11% vascular and 4% Lewy Body) was 3.4 months (SD = 3.27) before baseline. Table 2 presents distributions for all with dementia, including 52 without a carer or whose carer did not participate (therefore not in Table 1). There were differences in baseline characteristics between those who did or did not participate at T3 (Tables 1 and 2). Compared to those who dropped out, T3 participants were more likely to live in the North-East, fewer carers were in routine occupation, more people with dementia were homeowners and had higher baseline MMSE scores and there was a longer time between baseline and C19 interviews. There were no differences in baseline QoL scores by attrition.

**Table 1:** Distributions of socio-demographic variables and quality of life for the carer and the person with dementia they cared for at the DETERMIND baseline

	*n*	Baseline, all %/mean (SD)	*n*	Baseline, participated in Time 3%/mean (SD)	Difference by Time 3 participation^a^
Location	206		63		
Sussex		47		44	*P* = 0.02
North-East		26		38	
London		27		17	
Age at baseline, carer	206	66.5 (13.9)	63	66.2 (13.2)	*P* = 0.84
Female, carer	206	69	63	69	*P* = 0.85
Married, carer	205	82	63	81	*P* = 0.72
Education, carer	198		62		
No qualification		12		10	*P* = 0.80
Lower secondary school (O-level/GCSE)		26		23	
Upper secondary school (A/AS level)/vocational degree (NVQ 1–4 levels)		33		36	
Higher education degree		29		31	
Occupational class, carer	183		56		
Professional		43		45	*P* = 0.00
Intermediate		33		46	
Routine		25		9	
Home owner, carer	206	79	63	84	*P* = 0.24
Rural, carer (vs. urban)	203	15	62	13	*P* = 0.62
IMD, carer (higher = less deprived)	202	6.6 (2.8)	60	6.8 (2.8)	*P* = 0.48
Coresident with person with dementia	206	67	63	73	*P* = 0.22
Age at baseline, person with dementia	204	80.3 (8.3)	62	79.4 (8.2)	*P* = 0.29
Women with dementia	206	55	63	56	*P* = 0.97
Married, person with dementia	206	60	63	70	*P* = 0.06
Education, person with dementia	186		55		
No qualification		32		35	*P* = 0.58
Lower secondary school (O-level/GCSE)		29		22	
Upper secondary school (A/AS level)/Vocational degree (NVQ 1–4 levels)		23		25	
Higher education degree		16		18	
Occupational class, person with dementia	199		59		
Professional		37		41	*P* = 0.62
Intermediate		27		22	
Routine		36		37	
Home owner, person with dementia	206	72	63	84	*P* = 0.02
Rural, person with dementia (vs. urban)	206	11	63	8	*P* = 0.33
IMD, person with dementia (higher = less deprived)	206	6.3 (2.9)	63	6.3 (3.0)	*P* = 0.36
CDR score, T1	197	0.8 (0.5)	62	0.8 (0.5)	*P* = 0.71
CDR score, C19	103	1.0 (0.6)	47	1.0 (0.5)	*P* = 0.21
CDR score, T2	94	0.8 (0.4)	48	0.8 (0.5)	*P* = 0.76
CDR score, T3	62	1.3 (0.8)	59	1.2 (0.8)	*P* = 0.23
No of months since the diagnosis of dementia at baseline	181	3.4 (3.3)	54	3.6 (1.9)	*P* = 0.52
No of months between baseline and C19 interview	113	7.7 (1.8)	63	8.4 (1.8)	*P* = 0.00
C-DEMQQL total score	120	103.2 (1.7)	59	102.9 (2.4)	*P* = 0.91
C-DEMQOL subscales					
Meeting personal needs	124	20.5 (0.5)	61	19.9 (0.7)	*P* = 0.49
Carer well-being	125	19.9 (0.4)	62	20.1 (0.7)	*P* = 0.77
Carer–patient relationship	126	23.9 (0.3)	61	23.7 (0.5)	*P* = 0.67
Confidence in future	124	18.2 (0.4)	60	18.4 (0.6)	*P* = 0.86
Feeling supported	89	19.9 (0.6)	45	20.3 (0.8)	*P* = 0.68
DEMQOL-Proxy total score	142	88.5 (1.3)	63	89.7 (1.8)	*P* = 0.60
DEMQOL-Proxy subscales					
Feelings	142	27.5 (0.5)	63	27.4 (0.9)	*P* = 0.94
Memory	142	26.0 (0.5)	63	26.1 (0.7)	*P* = 0.94
Everyday life	142	35.1 (0.5)	63	36.3 (0.6)	*P* = 0.18

CDR, Clinical Dementia Rating ; IMD, index of multiple deprivation.

^a^Difference tests between those who participated versus did not participate in Time 3 interview showing *P* values for chi square for categorical variables and *t* test for means.

**Table 2:** Distributions of person with dementia socio-demographic variables and quality of life at the DETERMIND baseline

	*n*	Baseline all %/mean (SD)	*n*	Baseline, participated in Time 3%/mean (SD)	Difference by Time 3 participation^a^
Location	262		69		
Sussex		47		52	*P* = 0.02
North-East		24		32	
London		28		16	
Carer/person with dementia interview	258		68		
Person with dementia only		20		25	*P* = 0.07
Non-coresident carer and person with dementia		26		16	
Coresident carer and person with dementia		53		59	
Age at baseline, person with dementia	252	80.2 (8.1)	68	78.8 (7.8)	*P* = 0.10
Women with dementia	254	56	68	53	*P* = 0.57
Married, person with dementia	254	52	68	59	*P* = 0.21
Education, person with dementia	233		62		
No qualification		31		29	*P* = 0.98
Lower secondary school (O-level/GCSE)		27		29	
Upper secondary school (A/AS level)/Vocational degree (NVQ 1–4 levels)		24		24	
Higher education degree		18		18	
Occupational class, person with dementia	244		62		
Professional		40		39	*P* = 0.85
Intermediate		26		29	
Routine		34		32	
Home owner, person with dementia	254	73	68	84	*P* = 0.02
Rural, person with dementia (vs. urban)	255	12	68	10	*P* = 0.66
IMD, person with dementia (higher = less deprived)	255	6.3 (2.8)	68	6.7 (2.8)	*P* = 0.16
MMSE score baseline, person with dementia	259	22.5 (5.1)	69	24.4 (3.7)	*P <* 0.00
No of months since the diagnosis of dementia at baseline	218	3.5 (3.2)	54	3.7 (2.0)	*P* = 0.55
No of months between baseline and C19 interview	140	7.7 (1.8)	69	8.6 (2.0)	*P* = 0.00
DEMQOL total score	177	87.2 (1.0)	68	88.0 (1.7)	*P* = 0.64
DEMQOL subscales					
Feelings	178	37.6 (0.5)	69	38.1 (0.5)	*P* = 0.61
Memory	178	18.9 (0.3)	68	19.0 (0.5)	*P* = 0.76
Everyday life	176	30.8 (0.3)	68	31.0 (0.6)	*P* = 0.77

IMD, index of multiple deprivation; MMSE, Mini-Mental State Examination.

^a^Difference tests between those who participated vs. did not participate in Time 3 interview showing *P*-values for chi square for categorical variables and t-test for means.

The data suggest decline in QoL in carers (Table 3) and some increases in QoL in people with dementia (Table 4). In terms of performance, all three total QoL scores (C-DEMQOL, DEMQOL, DEMQOL-Proxy) showed high internal consistency (Cronbach’s alphas 0.90 or higher for all time points) and QoL subscales showed adequate internal consistence (0.72–0.94, Tables 3 and 4).

**Table 3:** Observed means (standard deviations, SD) and latent growth curve (LCG) unstandardised estimates (standard errors, SE) for carer quality of life in four waves of DETERMIND

	Observed mean scores for all participants, (SD)								LCG estimates, (SE)			
	*n*	Time 1 (T1)	*n*	C19	*n*	Time 2 (T2)	*n*	Time 3 (T3)	Intercept	Slope 1 T1-C19	Slope 2 C19-T3	Slope 3 T2-T3
C-DEMQQL total score	179	103.1 (18.2)	103	97.8 (17.2)	99	102.1 (18.5)	61	98.4 (18.5)	103.1^*^^*^^*^ (1.3)	−4.2^*^^*^^*^ (1.2)	−0.6 (0.9)	−3.1 (2.1)
C-DEMQOL subscales												
Meeting personal needs	185	20.3 (5.4)	106	19.9 (5.4)	99	19.7 (5.9)	64	18.0 (5.1)	20.3^*^^*^^*^ (0.4)	0.0 (0.4)	−1.0^*^^*^^*^ (0.3)	−1.7^*^^*^ (0.5)
Carer well-being	187	19.9 (4.9)	104	19.6 (4.9)	99	20.2 (4.8)	64	18.5 (5.2)	19.9^*^^*^^*^ (0.4)	−0.1 (0.4)	−0.8^*^^*^ (0.3)	−1.9^*^^*^^*^ (0.5)
Carer–patient relationship	187	23.8 (3.5)	105	23.3 (3.7)	100	23.6 (3.7)	64	23.1 (3.7)	23.9^*^^*^^*^ (0.3)	−0.5^*^ (0.3)	−0.0 (0.2)	−0.3 (0.4)
Confidence in future	184	18.3 (4.6)	104	17.8 (5.0)	100	19.3 (5.0)	61	18.0 (5.1)	18.3^*^^*^^*^ (0.3)	−2.7^*^^*^^*^ (0.9)	0.9^*^^*^ (0.3)	−0.5 (0.6)
Feeling supported	134	20.1 (5.3)	89	17.3 (4.2)	80	19.0 (5.4)	50	19.2 (5.2)	20.0^*^^*^^*^ (0.4)	−2.6^*^^*^^*^ (0.5)	0.9^*^^*^ (0.3)	0.8 (0.8)

Cronbach’s alphas for Time 1, C19, Time 2 and Time 3: 0.92, 0.93, 0.93, 0.93 (C-DEMQQL total score), 0.91, 0.93, 0.94, 0.90 (C-DEMQOL Meeting personal needs), 0.86, 0.85, 0.83, 0.87 (C-DEMQOL Carer wellbeing), 0.74, 0.76, 0.80, 0.77 (C-DEMQOL Carer-patient relationship), 0.81, 0.87, 0.87, 0.86 (C-DEMQOL Confidence in future), 0.81, 0.82, 0.78, 0.82 (C-DEMQOL Feeling supported).

^*^ *P* < 0.05.

^*^^*^ *P* < 0.01.

^*^^*^^*^ *P* < 0.001.

**Table 4:** Observed means (standard deviations, SD) and latent growth curve (LCG) unstandardised estimates (standard errors, SE) for quality of life in people with dementia in four waves of DETERMIND

	Observed mean scores for all participants, (SD)								LCG estimates, (SE)			
	*n*	Time 1 (T1)	*n*	C19	*n*	Time 2 (T2)	*n*	Time 3 (T3)	Intercept	Slope 1 T1–C19	Slope 2 C19–T3	Slope 3 T2–T3
DEMQOL total score	245	87.4 (13.2)	91	87.1 (13.2)	99	87.9 (14.2)	68	90.6 (13.1)	87.3^*^^*^^*^ (0.8)	0.0 (1.0)	1.1 (0.7)	2.4^*^ (1.2)
DEMQOL subscales												
Feelings	247	37.7 (7.0)	92	37.2 (7.2)	99	37.2 (7.6)	69	37.9 (7.1)	37.7^*^^*^^*^ (0.4)	−0.4 (0.5)	0.0 0.4)	0.4 (0.7)
Memory	246	18.9 (3.9)	91	19.1 (4.1)	98	19.6 (4.3)	67	20.5 (3.7)	18.9^*^^*^^*^ (0.2)	0.2 (0.3)	0.7^*^^*^ (0.2)	1.0^*^ (0.4)
Everyday life	244	30.9 (4.5)	91	30.8 (5.2)	99	31.1 (4.9)	66	32.0 (4.2)	30.8^*^^*^^*^ (0.3)	0.0 (0.5)	0.5 (0.3)	1.0^*^ (0.5)
DEMQOL-Proxy total score	205	88.9 (14.6)	110	88.4 (15.5)	102	89.3 (14.3)	63	95.3 (13.6)	88.9^*^^*^^*^ (1.0)	−0.1 (1.1)	2.3^*^^*^ (0.8)	3.8^*^^*^ (1.4)
DEMQOL-Proxy subscales												
Feelings	205	27.4 (6.4)	110	26.7 (6.2)	103	26.4 (6.7)	64	27.7 (5.1)	27.5^*^^*^^*^ (0.4)	−0.9 (0.5)	0.4 (0.3)	1.2^*^ (0.6)
Memory	205	26.0 (5.9)	110	25.8 (6.7)	102	26.7 (6.2)	63	29.0 (6.0)	26.0^*^^*^^*^ (0.4)	0.1 (0.5)	1.2^*^^*^ (0.4)	1.6^*^^*^ (0.6)
Everyday life	205	35.5 (5.8)	110	36.0 (5.9)	102	36.6 (5.1)	63	38.4 (5.3)	35.5^*^^*^^*^ (0.4)	0.6 (0.4)	0.8^*^ (0.4)	1.3^*^ (0.6)

Cronbach’s alphas for Time 1, C19, Time 2 and Time 3: 0.91, 0.90, 0.92, 0.92 (DEMQOL total score), 0.85, 0.85, 0.87, 0.83 (DEMQOL Feelings), 0.81, 0.82, 0.87, 0.87 (DEMQOL Memory), 0.81, 0.83, 0.83, 0.81 (DEMQOL Everyday life), 0.91, 0.91, 0.90, 0.91 (DEMQOL-Proxy total score), 0.86, 0.83, 0.87, 0.78 (DEMQOL-Proxy Feelings), 0.85, 0.89, 0.87, 0.89 (DEMQOL-Proxy Memory), 0.80, 0.81, 0.74, 0.81 (DEMQOL-Proxy Everyday life).

^*^ *P* < 0.05.

^*^^*^ *P* < 0.01.

^*^^*^^*^ *P* < 0.001.

### **Change in** QoL

Scores and LGC intercepts and slopes are shown in Table 3 for carers and Table 4 for people with dementia. Slope estimates showed the overall carer QoL dropped between baseline and C19 interviews (Table 3). This decline in carer QoL was mostly due to diminished ‘Confidence in future’, ‘Feeling supported‘ and ‘Carer-patient relationship’. There was a subscale-specific decline in ‘Meeting personal needs’ and ‘Carer wellbeing’ between C19–T3 and T2–T3. ‘Confidence in future’ and ‘Feeling supported’ increased between C19 and T3. None of the carer QoL items returned to baseline levels by T3.

For people with dementia, there was no change in QoL measures between T1 and C19 (Table 4). Their QoL increased between T2 and T3 due to increases in subscales ‘Memory’ and ‘Everyday life’. QoL related to ‘Memory’ also increased between C19 and T3. A similar, stronger pattern was seen using DEMQOL-Proxy; total score increased after C19 interview; all subscales, but especially ‘Memory’, contributed to the increase. All person with dementia QoL scores were higher at T3 than baseline levels. All residual variances for levels and slopes in carers and people with dementia were significant (*P* < 0.001), suggesting direction and speed of change varied between individuals.

The fully adjusted estimates for means are shown in Supplementary Tables 1 and 2. The observed means and the *n* for each QoL measure by wave are in Tables 3 and 4. Supplementary Figures 1a–4b illustrate observed and estimated means over the four waves (see Supplementary text 1 for attrition).

### Change in QoL by location and other characteristics

Results for adjusted models with all covariates are shown in Supplementary Tables 3–8 for carers and 9–16 for people with dementia. Carers in rural locations experienced a faster decrease in QoL between T1–C19 interviews and a faster increase in QoL between T2–T3 (Supplementary Table 3). This was mostly due to decline in ‘Carer wellbeing’ between T1–C19 (Supplementary Table 5) and increase in ‘Feeling supported’ in later interviews (Supplementary Table 8). For people with dementia, rural/urban location was not associated with levels or change in QoL. Few other background characteristics were associated with the level or change in QoL (see Supplementary Tables 3–16). Low CFI values (<0.90) in some models suggest the combination of little overall change and weak predictors produced a low specification of the model.

## Discussion

This is the first study to longitudinally map QoL trajectories of people with dementia and their carers over a 3-year period, using data collected before, during and after the pandemic. This allowed for an exploration of the short- and longer-term impacts of the pandemic on QoL. We found that people with dementia maintained their QoL during the pandemic, and even experienced some increase towards the end of the period. However, their family carers had a general deterioration in their QoL over the same period and this is of concern. The decline was sharpest during the first lock-down and driven by ‘Confidence in Future,’ ‘Feeling supported’ and ‘Care-patient relationship’ subscales of C-DEMQOL. ‘Confidence in future’ and ‘Feeling supported’ were the only subscales to show some recovery over the pandemic (C19–T3). The removal of public health restrictions and gradual re-introduction of informal and formal post-diagnostic care services may have provided some needed and valued support but not enough to recover carers’ overall QoL post-pandemic.

Before discussing our findings, we must consider the important limitations of our study. First, although DETERMIND is diverse, it was not drawn to be representative of the UK population of people with dementia and their carers. When compared with national projections, our cohort may have been diagnosed at a later age with a higher percentage of the participants being men and a higher percentage of the carers women and from professional occupations (see Table 1) [25]; for a detailed discussion of representativeness see Supplementary text 1. Second, not all eligible people agreed to participate in the C19 data collection and there was high drop-out post-pandemic even when face-to-face data collection resumed. To address this, we included people with partial information and full information maximum likelihood estimation took into account patterns of missingness. The initial non-response (people with no data) and possible unobserved factors may have caused bias, although our statistical modelling is likely to have mitigated a number of these issues (see Supplementary Text 1 for detailed discussion of attrition). Third, we focused on QoL, a broad measure of overall well-being widely used in research and policy [5]. Although we measured this using a dementia-specific QoL tool, our approach did not enable us to investigate more subtle changes in psychological function, cognition, clinical depression or anxiety. However, we were partially able to address this and overcome limitations in previous research, by examining the subscales of QoL within each measure and so provide a more nuanced picture of well-being.

Contextualising our results within the wider academic literature demonstrates how they support and build on previous studies, enabling some conclusions to be drawn. Our finding that generally people with dementia maintained their levels of QoL over this period aligns with previous research [13, 14] and provides more substantial support given we collected data directly from people with dementia using a dementia-specific QoL measure. It is an important finding that decline in QoL is not inevitable following the onset and diagnosis of dementia, even during a global pandemic. The increase in QoL observed later (T2–T3) was particularly in the subscales of ‘Memory’ and ‘Everyday life’. The environmental changes, which resulted from an easing of the Covid restrictions, may have had influence in improving these QoL scores for people with dementia, driven by an increase in people’s feelings of positivity towards everyday life and a reduction in their worries regarding their memory. This finding supports calls to understand dementia holistically through a biopsychosocial lens and so acknowledge the importance of the environmental context on people’s lived experiences [1]. Adopting this lens ensures we seek to support people to live well with dementia through enabling social inclusion and promoting mental, physical and social stimulation rather than solely focusing on biomedical challenges [40].

Our finding that family carer QoL deteriorated during the pandemic is consistent with previous research [24, 25] and international studies that report the difficulties carers encountered during this period [22]. However, it is of concern to find that carer QoL generally continued to decline even towards the end of the pandemic. This is not the pattern seen in longitudinal studies conducted pre-pandemic that show carer QoL can be maintained over a period of 18 months to 3 years post-diagnosis [15–18]. Although cohort effects may play a role, there is the potential that our observed long-term detrimental impact to carer QoL may be a result of the pandemic. We found the most notable declines were in carers’ abilities for ‘Meeting the personal needs of the person with dementia’ and ‘Carer well-being.’ These may be addressed through better post-diagnostic support targeted specifically at family carers of people with dementia. Carers play an invaluable role in the lives of people with dementia and wider society, equating to substantial monetary savings for society [41, 42]. Our findings suggest that, post-pandemic, they may require greater support to maintain their QoL.

We found rural/urban differences in QoL trajectories of carers, with the rural location associated with a faster decline in carer QoL during the pandemic (baseline–C19) and a greater recovery towards the end of the follow-up (T2–T3). Research on the geo-socio-political rural environment on dementia experience shows carers and people with dementia can rely on tightknit rural informal support networks to mitigate some of the challenges associated with a lack of formal care services [43–45]. It is possible that during the pandemic social distancing policies led to a reduction in these valuable informal support networks for rural carers, resulting in faster decline in carer QoL, which only recovered once restrictions were lifted. Further research is needed to elucidate the influence of rurality on the dementia experience both during and post-pandemic.

It is important to acknowledge the great variation in individual QoL change scores for people with dementia and family carers, with some reporting large changes over this time period whereas others reported only minimal changes (both positive and negative). This finding emphasises the differences in people’s experiences of living with dementia and managing the challenges of the pandemic. Qualitative research has highlighted how some people with dementia welcomed the lock-down and took sanctuary within the ‘shrinking world’ to come to terms with their dementia and restore their well-being [19, 23]. Other studies have also highlighted the importance of positive dyadic coping strategies for supporting people with dementia and their carers during this difficult period [46]. We intend to seek to unpick some of this variation by examining the impact of socio-demographic determinants, structural inequalities when accessing informal and formal services and individual and dyadic coping mechanisms on the post-diagnostic trajectories of people with dementia and their carers in the DETERMIND cohort.

## Conclusions

Our data show that, although there is variation in participants’ experiences, generally the major QoL impacts both during and after the pandemic have been on family carers of people with dementia rather than on people with dementia themselves. It is positive that, even during a global pandemic, a dementia diagnosis does not necessarily equate to deterioration in life quality, but it is a concern that carer QoL continued to decline even as restrictions were lifted and formal services re-opened. Our findings suggest that post-pandemic family carers may require better support to enable them to care well and to sustain their own well-being and that of the person with dementia.

## Supplementary Material

supplementary_materials_afad233
