# Supplementary material for: Long-term impact of the COVID-19 pandemic on the quality of life of people with dementia and their family carers
Source: Age Ageing. 2024 Jan 25;53(1):afad233. doi: 10.1093/ageing/afad233 (PMC10811518; doi:10.1093/ageing/afad233)
Supplement: supplementary_materials_afad233 [file supplementary_materials_afad233.zip › supplementary_materials_afad233/aa-23-1166-File002.docx]

**Supplementary Text 1.**

**Detailed discussion of sample characteristics, non-participation (all data missing) and representativeness of the sample at baseline**

The comparison of the baseline characteristics of DETERMIND with nationally representative figures is challenging given there are few statistics available that cover the same time period and target population (people with newly diagnosed dementia and their carers). Some information can be found on the gender and age profiles of people with dementia and their carers.

-***Gender:*** According to 2018-19 data in England [1,2], 65% of the people with dementia were women, and 63% of the carers of people with dementia were women, (any time from diagnosis). Using data from a large mental health care database linked to 2008–2016 English hospital data among people aged 65+, the proportion of women with a new dementia diagnosis was 55.4% [3]. In the current sample, 55% (sample with carers) and 56% (sample of all, some without carers) of the people with dementia and 69% of the carers were women.

-***Age:*** Based on care electronic health records including linked Hospital Episode Statistics and mortality data for adults aged ≥65 years between year 1997-2018, the average mean age at dementia diagnosis was 75.5 years for the White, 72.3 years for the South Asian and 73.1 years for the Black people in England [4]. In another study cited above using 2008–2016 English hospital data among people aged 65+, the mean age at dementia diagnosis was 82.2 years [3]. In the current study, the age of those with newly diagnosed dementia was 80.3 years (sample with carers) and 80.2 years (sample of all, some without carers).

The diagnosis rate of dementia was 68.6% in England in December 2019 [5]. The current sample is restricted to those who had a diagnosis. The study was carried out in three locations.

**Detailed discussion of our attrition over the follow-up (partial data missing) and missing data estimation in the latent growth models**

There were some differences in baseline characteristics between those who did or did not participate in the last wave (T3) (Tables 1 and 2). Compared to those who dropped out before T3, T3 participants were more likely to live in the North-East and less likely to live in London, fewer carers were in routine occupation, more people with dementia were homeowners and had higher baseline MMSE scores (indicating less severe dementia), and there was a longer time between baseline and C19 interviews. There were no differences in baseline quality of life (QoL) scores by attrition.

Attrition is known to be associated with lower socioeconomic status and poorer functioning and there are several methods to handle the missing information in longitudinal data [6]. In the current study, the above-mentioned differences between those who participate and those who drop out after baseline were taken into account using all available data (means and variances of all available data points) and estimating with full information maximum likelihood (FIML) [7]. This method is equivalent to multiple imputation [8]. Both methods are widely used to handle longitudinal attrition. FIML together with robust standard error estimation (MLR) provides reliable estimation when the dataset includes a representative set of socioeconomic and other background variables, even in cases when dataset is small and attrition over time is high [9,10]. FIML uses an expectation maximization algorithm to identify parameter estimates that maximize the model fit with the observed data, i.e. it minimizes the distance between the observed and predicted data [11]. The observed patterns in participation (e.g. lower socio-economic status in those who dropped out) had been used in the modelling.

FIML assumes missing at random (MAR) in handling missing data[7]. This means that the patterns of missingness are random after conditioning on observed factors in the data. It is possible that some missingness may be not at random [12,13], i.e. cannot be predicted using the information from the covariates and previous pattern of slope for QoL. The person may experience a sudden decline or increase in QoL which is not picked in the model. The existing evidence on the longitudinal quality of life in people with dementia suggests that quality of life does not decline over time [14,15]. The changes in quality of life may be slower and less dependent on deteriorating health as for instance direct functional measures such as ADL or IADL, which may show sudden drops especially before death [16]. These observations mean that a rapid, unpredictable decline or increase in QoL is unplausible and using alternative methods, based on missing not as random (MNAR), may not provide better estimates. Latent growth curve models fitted with the assumption of MAR have been found to be robust when missing data mechanisms (e.g. that lower occupational class is associated with missingness) is taken into account in the model [13]. The MNAR estimation relies on the assumptions of missingness, which are often untestable [9,17].

Our study shows that the associations of quality of life with higher socio-economic status was not always positive, but varied so that some associations were positive, some negative, and for many there were no associations. E.g. a higher educational status was associated with a faster increase in QoL after pandemic in people with dementia (Supplementary Table 9), but higher educational status of the carer was associated with lower quality of life among people with dementia in DEMQOL-proxy (Supplementary Table 13). Furthermore, home ownership of the person with dementia (which was associated with less attrition) was associated with a slower increase in QoL between T2 and T3 (Supplementary Table 15). In carers, a higher occupational class (which was associated with less attrition) was associated with a slower change of QoL subscale ‘Carer-patient relationship’ between C19 and T3 (Supplementary Table 6). However, in the same model, upper secondary education compared to no qualification was associated with lower initial ‘Carer-patient relationship’ score but faster change of it between T1 and C19.

These examples show that there was no uniform pattern in how socioeconomic background factors were associated with QoL and attrition. Any observed attrition after baseline due to background differences or differences in QoL was taken into account in the modelling. As the patterns varied by the background factors, the estimates that were produced by the models showed occasionally higher and occasionally lower point estimates (especially in T2 and T3 where the attrition was higher): compare the observed and estimated curves in Supplementary figures 1a and 1b, 2a and 2b, 3a and 3b, and 4a and 4b.

As was expected, the baseline level of severity of dementia was to some extent associated with attrition and poorer quality of life. However, similar to socio-economic background, the patterns were not consistent. For instance, the associations between severity dementia and QoL were not always significant (see Supplementary Tables 3-16), and although MMSE scores were lower among those who did not initially participate (Table 2), there was no difference in CDR by attrition in the dyads of carer and person with dementia (Table 1). Again, any differences due to attrition were taken into account using full information maximum likelihood in the models, as described above. Carer-rated QoL for people with dementia included people with dementia who were not able to participate themselves. This measure (DEMQOL-Proxy) showed similar stability as the self-rated QoL for people with dementia. The sample selection included people with newly diagnosed dementia, so most of the participants showed only mild or moderate cognitive impairment at baseline. As already discussed above, people with dementia generally do not experience a rapid decline of quality of life [14,15]. Using proxy measures ensures that the sample also include those people with dementia who may have experienced a rapid decline in functioning.

**References**

1. Alzheimer’s Research UK. The Impact of Dementia on Women. 2022.

2. Cebr. The economic cost of dementia to English businesses-2019 update. A report for Alzheimer’s Society. 2019.

3. Sommerlad A, Perera G, Singh-Manoux A *et al.* Accuracy of general hospital dementia diagnoses in England: Sensitivity, specificity, and predictors of diagnostic accuracy 2008–2016. *Alzheimer’s & Dementia* 2018;**14**:933–43.

4. Mukadam N, Marston L, Lewis G *et al.* Incidence, age at diagnosis and survival with dementia across ethnic groups in England: A longitudinal study using electronic health records. *Alzheimer’s and Dementia* 2023;**19**:1300–7.

5. Office for Health Improvement  & Disparities. Statistical commentary: dementia profile, March 2021 update - GOV.UK. 2022.

6. Little RJA, Rubin DB. *Statistical Analysis with Missing Data. Second Edition*. John Wiley & Sons, 2002.

7. Acock AC. Working with missing values. *Journal of Marriage and Family* 2005;**67**:1012–28.

8. Lee T, Shi D. A comparison of full information maximum likelihood and multiple imputation in structural equation modeling with missing data. *Psychol Methods* 2021;**26**:466–85.

9. Allison PD. Missing Data Techniques for Structural Equation Modeling. *J Abnorm Psychol* 2003;**112**:545–57.

10. Shi D, DiStefano C, Zheng X *et al.* Fitting Latent Growth Models with Small Sample Sizes and Non-normal Missing Data. *Int J Behav Dev* 2021;**45**:179.

11. McLachlan GJ, Krishnan T. The EM Algorithm and Extensions: Second Edition. 2007, DOI: 10.1002/9780470191613.

12. Jackson H, Engelman M, Bandeen-Roche K. Robust Respondents and Lost Limitations: The Implications of Nonrandom Missingness for the Estimation of Health Trajectories. *J Aging Health* 2019;**31**:685–708.

13. Lavikainen P, Leskinen E, Hartikainen S *et al.* Impact of missing data mechanism on the estimate of change: a case study on cognitive function and polypharmacy among older persons. *Clin Epidemiol* 2015;**7**:169–80.

14. King D, Farina N, Burgon C *et al.* Factors associated with change over time in quality of life of people with dementia: longitudinal analyses from the MODEM cohort study. *BMC Geriatr* 2022;**22**:1–13.

15. Clare L, Woods RT, Nelis SM *et al.* Trajectories of quality of life in early-stage dementia: individual variations and predictors of change. *Int J Geriatr Psychiatry* 2014;**29**:616–23.

16. Cohen-Mansfield J, Skornick-Bouchbinder M, Brill S. Trajectories of End of Life: A Systematic Review. *The Journals of Gerontology: Series B* 2018;**73**:564–72.

17. Enders CK. Missing not at random models for latent growth curve analyses. *Psychol Methods* 2011;**16**:1–16.
