# Supplementary material for: Long-term impact of the COVID-19 pandemic on the quality of life of people with dementia and their family carers
Source: Age Ageing. 2024 Jan 25;53(1):afad233. doi: 10.1093/ageing/afad233 (PMC10811518; doi:10.1093/ageing/afad233)
Supplement: supplementary_materials_afad233 [file supplementary_materials_afad233.zip › supplementary_materials_afad233/aa-23-1166-File003.docx]

***Supplementary Table 1: Latent Growth Curve model estimated means (Standard Errors, SE) for carer quality of life in four waves of DETERMIND, n = 206***

|  | Time 1  Mean (*SE*) | C19  Mean (*SE*) | Time 2  Mean (*SE*) | Time 3  Mean (*SE*) |
| --- | --- | --- | --- | --- |
| C-DEMQQL total score | 103.0 (1.3) | 98.6 (1.4) | 98.5 (1.6) | 99.5 (2.4) |
| C-DEMQOL subscales |  |  |  |  |
| Meeting personal needs | 20.3 (0.4) | 20.3 (0.4) | 19.4 (0.4) | 18.7 (0.6) |
| Carer wellbeing | 19.9 (0.4) | 19.8 (0.4) | 18.9 (0.4) | 18.2 (0.6) |
| Carer-patient relationship | 23.8 (0.3) | 23.4 (0.3) | 23.4 (0.3) | 23.5 (0.4) |
| Confidence in future | 18.3 (0.3) | 17.6 (0.4) | 17.8 (0.4) | 18.5 (0.5) |
| Feeling supported | 20.0 (0.4) | 17.3 (0.4) | 18.4 (0.4) | 19.6 (0.7) |

*** *p* < 0.001, ** *p* < 0.01, * *p* < 0.05. Models adjusted for study location, age and gender of carer and person with dementia, education, occupational class, home ownership, rural/urban location, for carer, coresidence with person with dementia, n of months since the diagnosis of dementia, n of month between baseline and C19 study, time-varying CDR scores.

***Supplementary Table 2: Latent Growth Curve model estimated means (Standard Errors, SE) for quality of life in people with dementia in four waves of DETERMIND***

|  | *n* | Time 1  Mean (*SE*) | C19  Mean (*SE*) | Time 2  Mean (*SE*) | Time 3  Mean (*SE*) |
| --- | --- | --- | --- | --- | --- |
| DEMQOL total score^a^ | 262 | 87.3 (0.8) | 86.8 (1.2) | 90.3 (1.0) | 93.7 (1.4) |
| DEMQOL subscales |  |  |  |  |  |
| Feelings^a^ | 262 | 37.7 (0.5) | 36.9 (0.7) | 38.0 (0.6) | 39.0 (0.8) |
| Memory^a^ | 262 | 18.9 (0.3) | 19.0 (0.4) | 20.2 (0.2) | 21.5 (0.4) |
| Everyday life^a^ | 262 | 30.8 (0.3) | 30.8 (0.5) | 32.0 (0.4) | 33.3 (0.5) |
| DEMQOL-Proxy total score^b^ | 206 | 89.0 (1.0) | 89.5 (1.2) | 90.9 (1.3) | 93.3 (1.9) |
| DEMQOL-Proxy subscales |  |  |  |  |  |
| Feelings^b^ | 206 | 27.5 (0.4) | 26.9 (0.6) | 27.1 (0.5) | 28.0 (0.8) |
| Memory^b^ | 206 | 26.0 (0.4) | 26.3 (0.6) | 27.4 (0.6) | 28.8 (0.8) |
| Everyday life^b^ | 206 | 35.5 (0.4) | 36.2 (0.4) | 36.3 (0.4) | 36.8 (0.8) |

*** *p* < 0.001, ** *p* < 0.01, * *p* < 0.05.

^a^ Models adjusted for study location, age, gender, education, occupational class, home ownership, rural/urban location, IMD (area deprivation) and MMSE score (cognition) at baseline for person with dementia, type of carer/person with dementia interview (coresidence), n of months since the diagnosis of dementia, n of month between baseline and C19 study.

^b^ Models adjusted for study location; age and gender of carer and person with dementia; education and occupational class for carer; home ownership, rural/urban location and IMD (area deprivation) for person with dementia; coresidence with person with dementia, n of months since the diagnosis of dementia, n of month between baseline and C19 study, time-varying CDR scores (Clinical Dementia Rating).
