# Supplementary material for: Long-term impact of the COVID-19 pandemic on the quality of life of people with dementia and their family carers
Source: Age Ageing. 2024 Jan 25;53(1):afad233. doi: 10.1093/ageing/afad233 (PMC10811518; doi:10.1093/ageing/afad233)

***Supplementary Table 3: The Associations of the background characteristics with Intercept and Slopes of carer quality of life (C-DEMQOL) total score in Latent Growth Curve (LCG) models, unstandardised estimates (Est.) and Standard Errors (SE)***


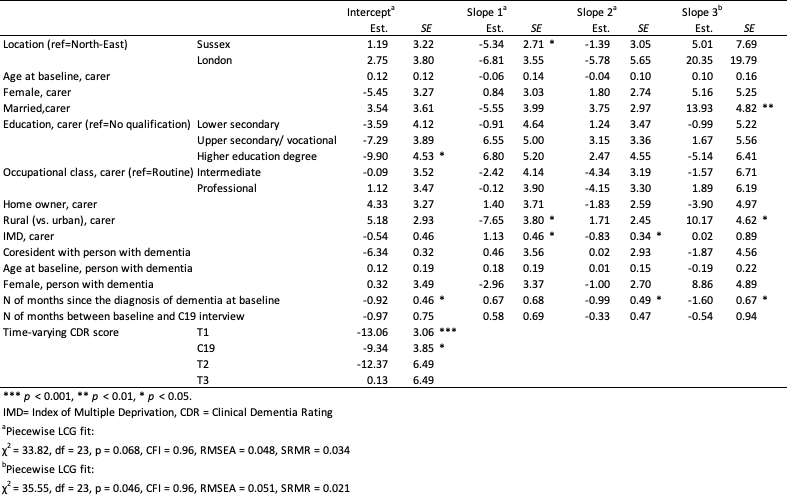


***Supplementary Table 4: The Associations of the background characteristics with Intercept and Slopes of carer quality of life (C-DEMQOL) subscale ‘Meeting personal needs’ in Latent Growth Curve (LCG) models, unstandardised estimates (Est.) and Standard Errors (SE)***


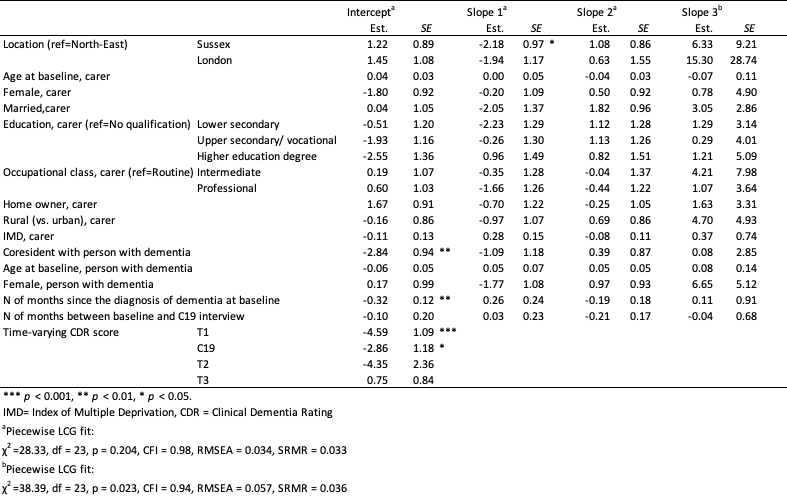


***Supplementary Table 5: The Associations of the background characteristics with Intercept and Slopes of carer quality of life (C-DEMQOL) subscale ‘Wellbeing’ in Latent Growth Curve (LCG) models, unstandardised estimates (Est.) and Standard Errors (SE)***


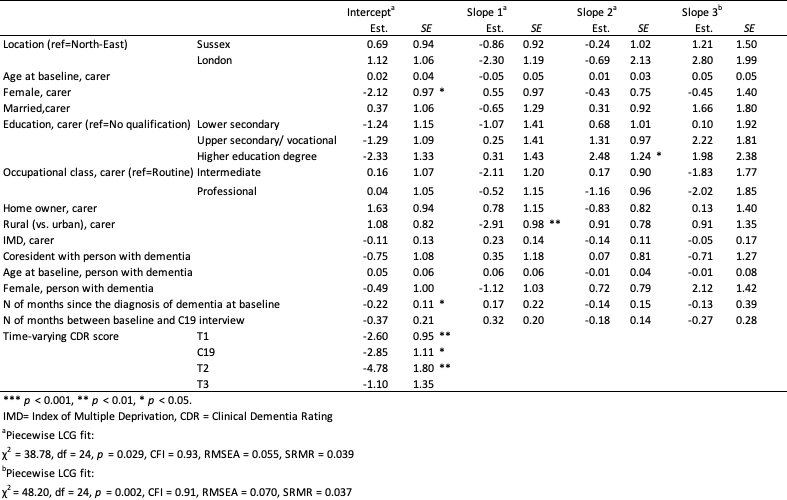


***Supplementary Table 6: The Associations of the background characteristics with Intercept and Slopes of carer quality of life (C-DEMQOL) subscale ‘Carer-patient relationship’ in Latent Growth Curve (LCG) models, unstandardised estimates (Est.) and Standard Errors (SE)***


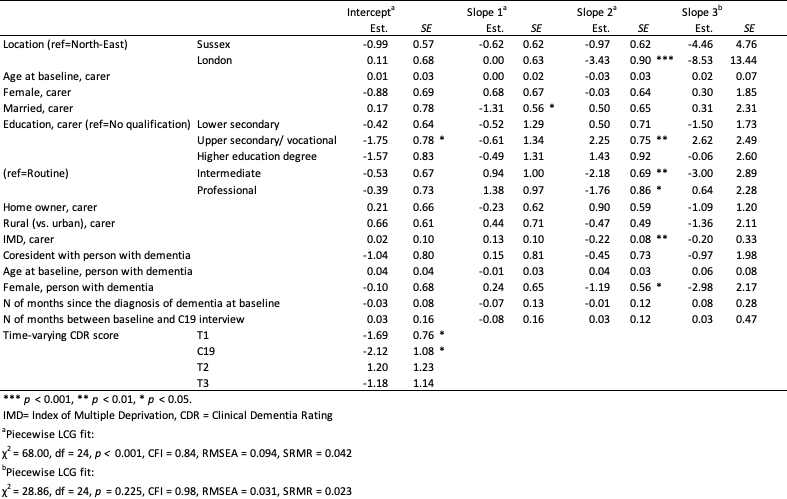


***Supplementary Table 7: The Associations of the background characteristics with Intercept and Slopes of carer quality of life (C-DEMQOL) subscale ‘Confidence in future’ in Latent Growth Curve (LCG) models, unstandardised estimates (Est.) and Standard Errors (SE)***

***
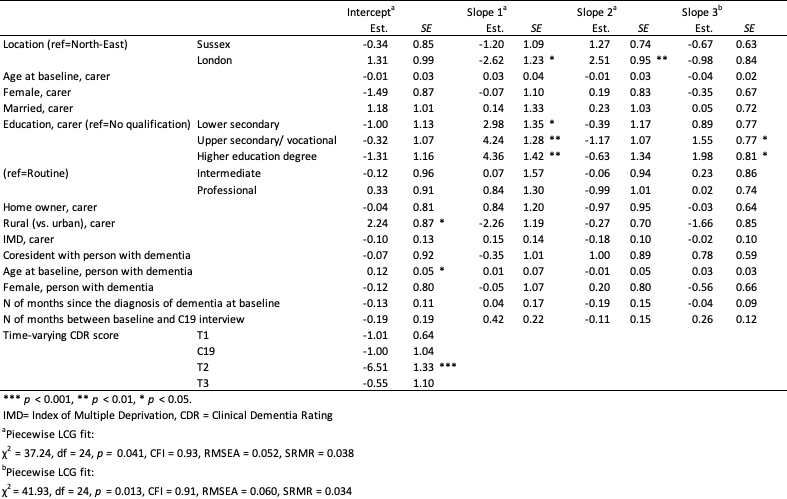
***

***Supplementary Table 8: The Associations of the background characteristics with Intercept and Slopes of carer quality of life (C-DEMQOL) subscale ‘Feeling supported’ in Latent Growth Curve (LCG) models, unstandardised estimates (Est.) and Standard Errors (SE)***


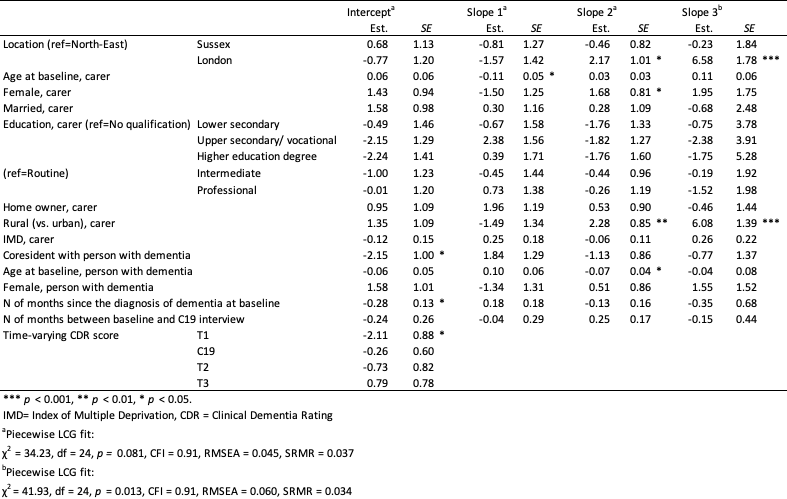

Supplement: supplementary_materials_afad233 [file supplementary_materials_afad233.zip › supplementary_materials_afad233/aa-23-1166-File004.docx]
