# Supplementary material for: Long-term impact of the COVID-19 pandemic on the quality of life of people with dementia and their family carers
Source: Age Ageing. 2024 Jan 25;53(1):afad233. doi: 10.1093/ageing/afad233 (PMC10811518; doi:10.1093/ageing/afad233)

***Supplementary Table 9: The Associations of the background characteristics with Intercept and Slopes of quality of life of person with dementia (DEMQOL) total score in Latent Growth Curve (LCG) models, unstandardised estimates (Est.) and Standard Errors (SE)***


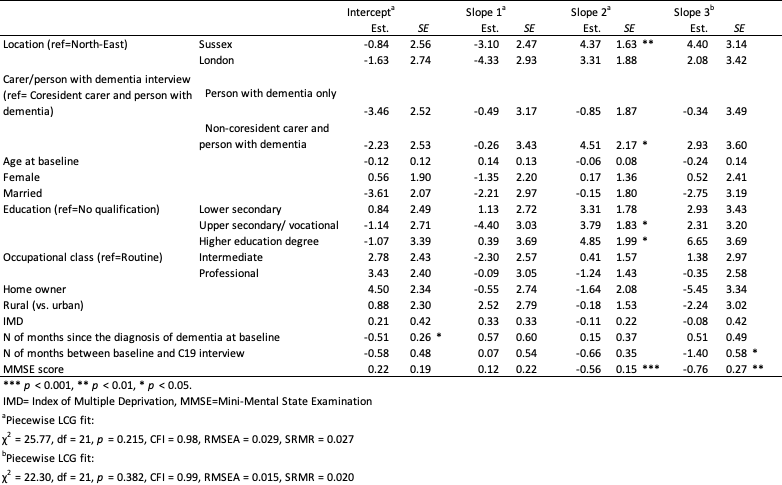


***Supplementary Table 10: The Associations of the background characteristics with Intercept and Slopes of quality of life of person with dementia (DEMQOL) subscale ‘Feelings’ total score in Latent Growth Curve (LCG) models, unstandardised estimates (Est.) and Standard Errors (SE)***


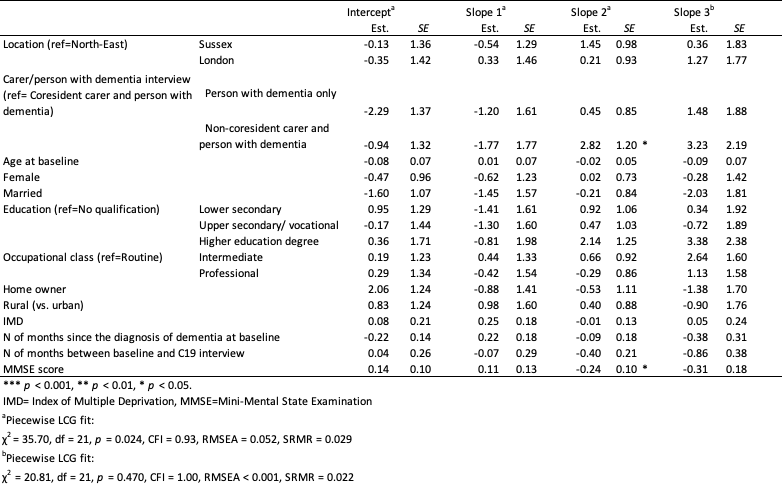


***Supplementary Table 11: The Associations of the background characteristics with Intercept and Slopes of quality of life of person with dementia (DEMQOL) subscale ‘Memory’ total score in Latent Growth Curve (LCG) models, unstandardised estimates (Est.) and Standard Errors (SE)***


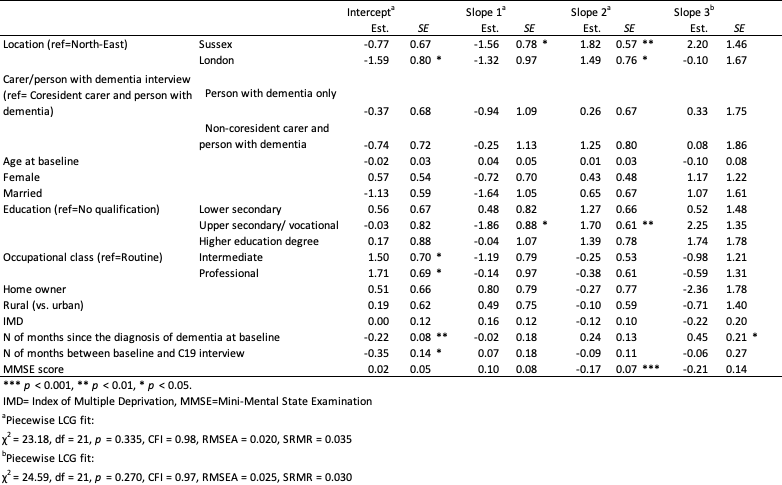


***Supplementary Table 12: The Associations of the background characteristics with Intercept and Slopes of quality of life of person with dementia (DEMQOL) subscale ‘Everyday life’ total score in Latent Growth Curve (LCG) models, unstandardised estimates (Est.) and Standard Errors (SE)***


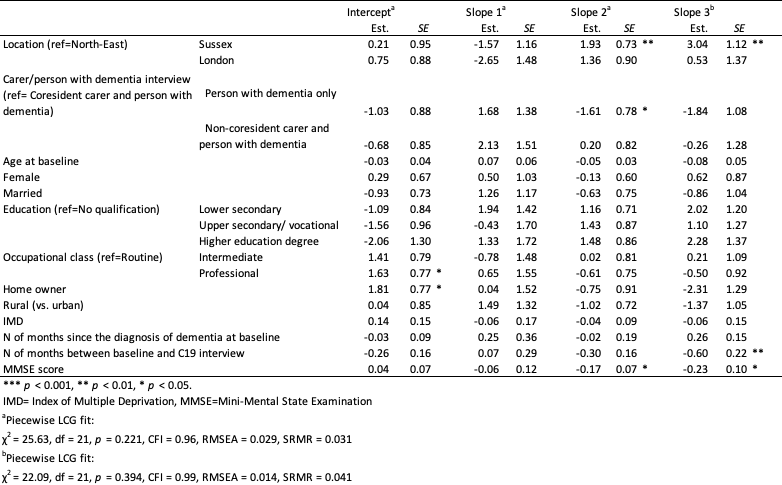


***Supplementary Table 13: The Associations of the background characteristics with Intercept and Slopes of carer assessed quality of life for person with dementia (DEMQOL-Proxy) total score in Latent Growth Curve (LCG) models, unstandardised estimates (Est.) and Standard Errors (SE)***


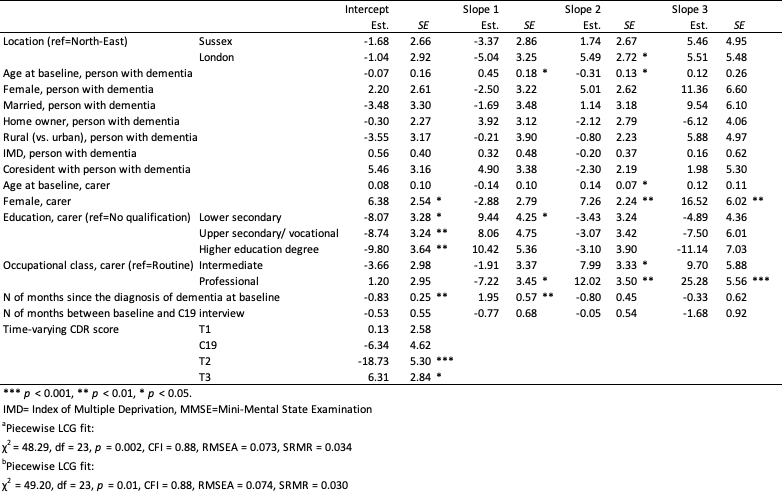


***Supplementary Table 14: The Associations of the background characteristics with Intercept and Slopes of assessed quality of life for person with dementia (DEMQOL-Proxy) subscale ‘Feelings’ in Latent Growth Curve (LCG) models, unstandardised estimates (Est.) and Standard Errors (SE)***

***
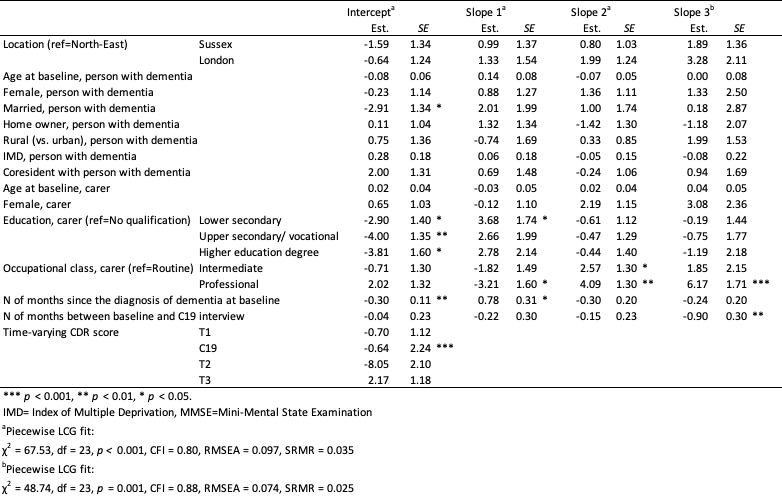
***

***Supplementary Table 15: The Associations of the background characteristics with Intercept and Slopes of carer assessed quality of life for person with dementia (DEMQOL-Proxy) subscale ‘Memory’ in Latent Growth Curve (LCG) models, unstandardised estimates (Est.) and Standard Errors (SE)***

***
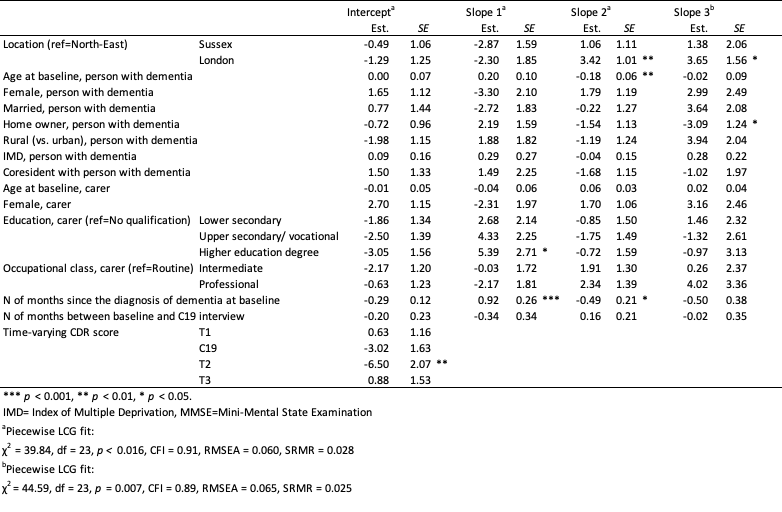
***

***Supplementary Table 16: The Associations of the background characteristics with Intercept and Slopes of carer assessed quality of life for person with dementia (DEMQOL-Proxy) subscale ‘Everyday life’ in Latent Growth Curve (LCG) models, unstandardised estimates (Est.) and Standard Errors (SE)***


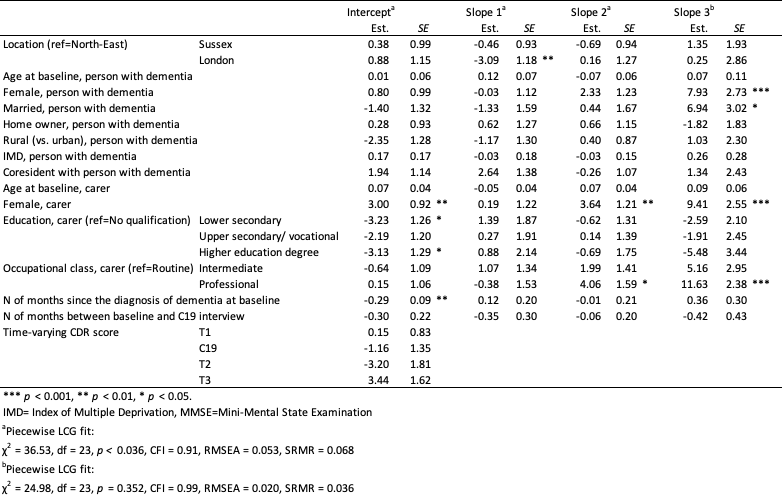

Supplement: supplementary_materials_afad233 [file supplementary_materials_afad233.zip › supplementary_materials_afad233/aa-23-1166-File005.docx]
