# Supplementary material for: Long-term impact of the COVID-19 pandemic on the quality of life of people with dementia and their family carers
Source: Age Ageing. 2024 Jan 25;53(1):afad233. doi: 10.1093/ageing/afad233 (PMC10811518; doi:10.1093/ageing/afad233)
Supplement: supplementary_materials_afad233 [file supplementary_materials_afad233.zip › supplementary_materials_afad233/aa-23-1166-File006.docx]

***Supplementary Figure 1a:*** Quality of life total scores for carers (CDEMQOL) and people with dementia (DEMQOL, DEMQOL-Proxy). The figure shows observed means from raw data (Table 3 and 4)

***Supplementary Figure 1b:*** Quality of life total scores for carers (CDEMQOL) and people with dementia (DEMQOL, DEMQOL-Proxy). The figure shows estimated means from the fully adjusted Latent Growth Model (Supplementary Table 3, 11 and 17)

***Supplementary Figure 2a:*** Quality of life (CDEMQOL) subscales for carers. The figure shows observed means from raw data (Table 3)

***Supplementary Figure 2b:*** Quality of life (CDEMQOL) subscales for carers. The figure shows estimated means from the fully adjusted Latent Growth Model (Supplementary Tables 4-8)

***Supplementary Figure 3a:*** Carer assessed quality of life (DEMQOL-Proxy) subscales for people with dementia. The figure shows observed means from raw data (Table 4)

***Supplementary Figure 3b:*** Carer assessed quality of life (DEMQOL-Proxy) subscales for people with dementia. The figure shows estimated means from the fully adjusted Latent Growth Model (Supplementary Tables 18-20)

***Supplementary Figure 4a:*** Quality of life (DEMQOL) subscales for people with dementia. The figure shows observed means from raw data (Table 4)

***Supplementary Figure 4b:*** Quality of life (DEMQOL) subscales for people with dementia. The figure shows estimated means from the fully adjusted Latent Growth Model (Supplementary Tables 12-14)
